# Fossil eggshell diversity of the Mussentuchit Member, Cedar Mountain Formation, Utah

**DOI:** 10.1371/journal.pone.0314689

**Published:** 2025-02-26

**Authors:** Joshua Hedge, Ryan T. Tucker, Peter J. Makovicky, Lindsay E. Zanno

**Affiliations:** 1 Department of Biological Sciences, North Carolina State University, Raleigh, North Carolina, United States of America; 2 Paleontology, North Carolina Museum of Natural Sciences, Raleigh, North Carolina, United States of America; 3 Department of Earth Sciences, Stellenbosch University, Stellenbosch, South Africa; 4 Department of Earth and Environmental Sciences, University of Minnesota, Minneapolis, Minnesota, United States of America; Universiteit Maastricht, NETHERLANDS, KINGDOM OF THE

## Abstract

The first fossil eggshell from the Cenomanian-age Mussentuchit Member of the Cedar Mountain Formation was described over fifty years ago. In the half-century since, oodiversity of this rock unit has been limited to a single, taxonomically unstable ootaxon, currently formulated as *Macroelongatoolithus carlylei*. Recently, there has been a renewed effort to recover and describe the macrofauna of the Mussentuchit; however, these advances are limited to the body fossil record. Here, we examine the range of eggshells present in the Mussentuchit Member and assess the preserved biodiversity they represent. Gross morphological and microstructural inspection reveals a greater diversity of eggshells than previously described. We identify six ootaxa: three Elongatoolithidae oogenera (*Macroelongatoolithus*, *Undulatoolithus*, *Continuoolithus*), eggs laid by oviraptorosaur dinosaurs; two oospecies of *Spheroolithus* laid by ornithopod dinosaurs; and *Mycomorphoolithus kohringi*, laid by a crocodylomorph. The diversity of Elongatoolithidae in the Mussentuchit requires a co-occurrence of at least three putative oviraptorosaurs, the oldest such phenomenon in North America. The occurrence of the crocodylomorph oogenus *Mycomorphoolithus* is the first recognized occurrence outside of Europe, and the youngest yet documented. This new ooassemblage is more representative of the known paleobiodiversity of Cenomanian-age strata of Western North America and complements the body fossil record in improving our understanding of this crucial—yet poorly documented—timeslice within the broader evolution of the Cretaceous Western Interior Basin.

## Introduction

The Early Cenomanian (99.94–98.9 Ma) Mussentuchit Member of the Cedar Mountain Formation is a renowned unit amongst vertebrate paleontologists studying the early Late Cretaceous [1]. Preserving an exceptionally diverse vertebrate fauna that now includes over 100 species [2, 3], this unit offers particularly valuable insight into the otherwise poor fossil record of the early Late Cretaceous, with abundant vertebrate microfossil assemblages of dinosaurs, mammals, crocodylomorphs, squamates, and turtles [2, 4–6]. Early diversity research focused on microvertebrate sampling. In the last decade, a growing diversity of new dinosaur taxa is documented by body fossils, including the allosauroid *Siats meekerorum* [7], the small tyrannosaur *Moros intrepidus* [8], and the rhabdodontomorph *Iani smithi* [9]. As one of the best-preserved records of paleobiodiversity in the early Late Cretaceous worldwide, this unit offers a window into the Western Interior Basin of North America in the run-up to the Cretaceous Thermal Maximum, the subsequent scarce terrestrial macrovertebrate fossil record in the Turonian, Coniacian, and Santonian [10–14], and the dinosaur diversification into the Campano-Maastrichtian when we see peak diversity [15, 16].

**Fig 1:**
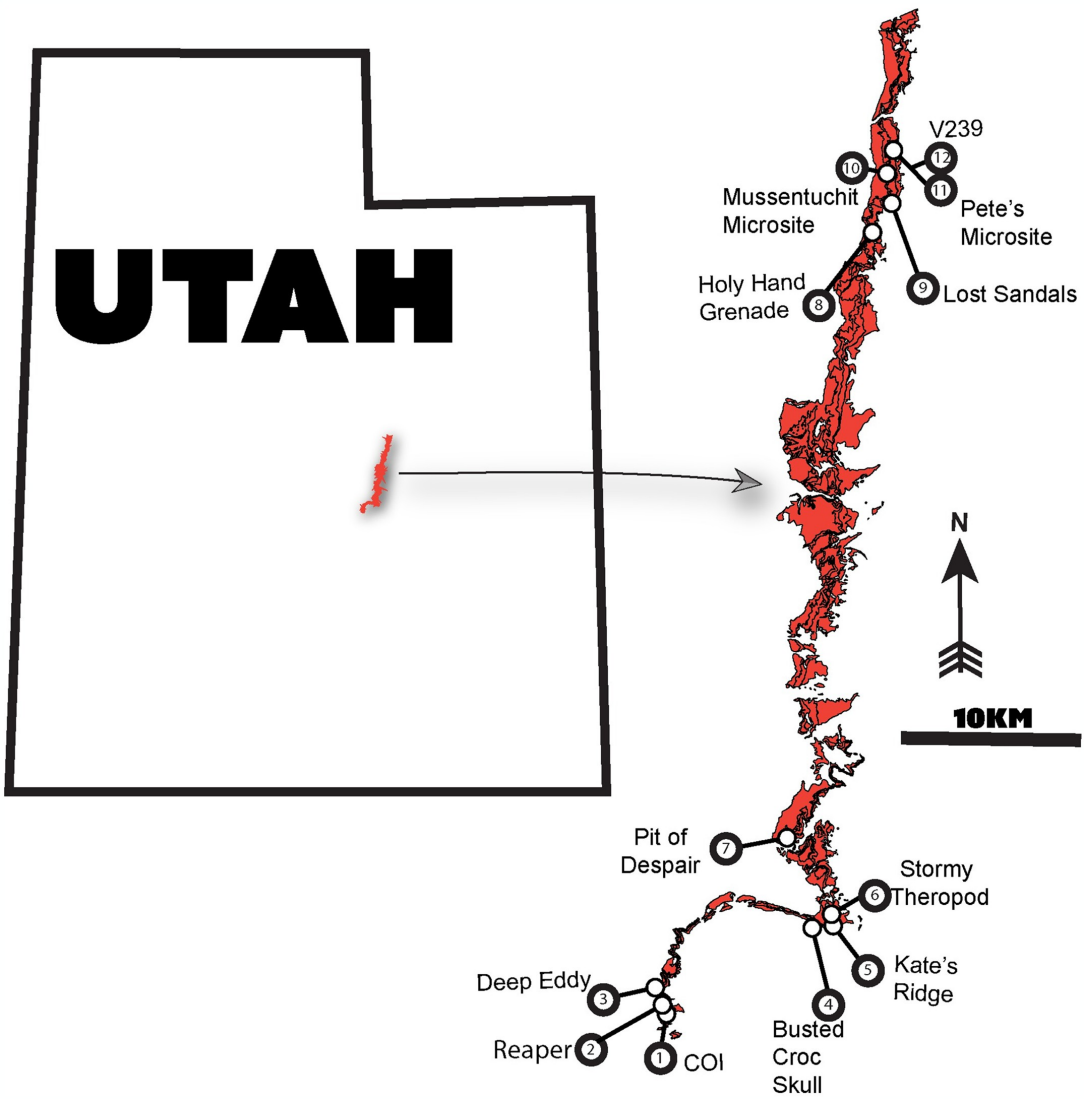
Location and geology of the study area. A) Map of present-day Utah with overlaid surface geology. The studied outcrop from the Mussentuchit is highlighted in yellow. Adapted from Utah Geological Survey B) outcrop of Mussentuchit Member from inset, with eggshell-bearing localities numbered.

The mid-Cretaceous is vital in terms of dinosaur paleobiogeography due in no small part to the influx of fauna into western North America that were previously endemic to Asia (e.g. [2, 8, 17, 18]. The entire region was ephemerally connected, via Beringia, to what is the modern-day Asian continent, supporting a recognized network of faunal exchange, the Early Cretaceous Laurasian Interchange Event (EKLInE) [19]. There is a documented turnover of western North American dinosaur faunas, and previously abundant clades were superseded by taxa from Asia. These taxa comprise a range of dinosaur clades first seen across the Western Interior Basin during the mid-Cretaceous, including: hadrosauriforms [9, 20, 21]; possible pachycephalosaurs [22]; ceratopsians [23]; and oviraptorosaurs [24]. The Mussentuchit Member encapsulates a microcosm of this turnover, such as the co-occurrence of the diminutive early tyrannosaur taxon *Moros intrepidus* with the Mussentuchit apex predator *Siats meekerorum* [8], one of the last allosauroid taxa in North America. Although previous efforts have contributed impressively to our collective knowledge of the paleoecology, paleoenvironment, and paleobiogeography of the early Late Cretaceous, previous work has yet to incorporate the new fossil eggshell recovered from the Mussentuchit Member into the makeup of this pivotal timeslice, and adjudge its implications for the broader Cretaceous Period. Since 2012, field crews from the Paleontology Lab at the North Carolina Museum of Natural Sciences (NCMNS) have collected an abundance of oolithic material from multiple localities in central Utah (Fig 1), ranging from >2,500 surface eggshell fragments to a clutch of 10+ *Macroelongatoolithus carlylei* eggs [18], referable to the largest known oospecies in the dinosaur fossil record [25].

We compare the eggshell specimens in the Mussentuchit Member to those of other North American units, including other lower Upper Cretaceous formations and the more prolific Late Cretaceous ooassemblages across the Western Interior Basin. We determine putative taxonomic affinities for the Mussentuchit ootaxa, and incorporate these into our existing knowledge of the Mussentuchit Member faunal composition. By filling gaps in the body fossil faunal data with fossil eggshell remains, we aim to better constrain the paleobiological diversity, and garner a better spatiotemporal understanding of Western Interior Basin faunistics during the Late Cretaceous Period.

### History of Mussentuchit Member ootaxonomy

Although it has changed names repeatedly, only one ootaxon was recognized from the Mussentuchit Member of the Cedar Mountain Formation for the past half century. The first ootaxon named from the member—*Oolithes carlylensis*—was described over fifty years ago [26]. At the time it represented the first described North American oospecies. The diagnosis was based on a rudimentary, then current existing taxonomic system [27, 28] that had been applied to Asian eggs. A more comprehensive parataxonomic system was adopted shortly after [29] and this led to the reassignment of BYU E-200 to the oogenus *Macroolithus*, within Elongatoolithidae, based on diagnostic features of microstructural layering. However, this reassignment was not broadly accepted, even after the wider adoption of this ootaxonomic system outside of Asia [30, 31].

Decades later, Jensen’s original material was then reinterpreted as referable to Spheroolithidae, and assigned to the oogenus *Boletuoolithus carlylensis* [31], based on an interpretation of prolatospherulitic shell units and pronounced radial ultrastructure diagnostic to Early Cretaceous eggshell. This material was subsequently reassessed by Zelenitsky and colleagues [32], who thin-sectioned both Jensen’s material and eggshell from four new localities within the Mussentuchit (n = 91) and concluded that all of the eggshell sampled belonged to *Macroelongatoolithus carlylei*. The study of Zelenitsky and colleagues was the first to recognize that these were eggshells with the ‘ornithoid-ratite’ morphotype [30] laid by theropods. Zelenitsky et al. [32] recognized that these eggshells were indistinguishable from colossal oviraptorosaur eggs discovered in Henan Province of China [33]. They further revised the specific epithet based on etymological aberration. Finally, Zelenitsky et al. [32] considered the oospecies of eggs from China (*M*. *xixiaensis*) a junior synonym of *M*. *carlylei* based on publication priority, until such a time that whole eggs from Utah were found and a true comparison could be made. Despite this diagnosis, the use of a single oogenus and oospecies has not always been widely adopted in the literature, and many other ootaxa including *M*. *xixiaensis* and other new *Macroleongatoolithus* oospecies *(M*. *zhangi* [34]; *M*. *goseongensis* [35]) were named from sites across Asia. Wang et al. [36] erected the oofamily Macroelongatoolithidae and included *Macroleongatoolithus* and the oogenera *Megafusoolithus* and *Longiteresoolithus* [34]. The discovery of a pair of colossal oviraptorosaur eggs from the Wayan Formation of Idaho [25], a unit similar in age to the holotype locality of *M*. *carlylei*, prompted reconsideration of the number of oospecies and oogenera within the taxon Macroelongatoolithidae. Simon et al. [25] discerned that the significant overlap of microstructure within all the aforementioned large taxa was sufficient to justify a single oogenus and oospecies for all these eggs, and to include them within the oofamily Elongatoolithidae. Thus, they synonymized all existing oogenera and oospecies with *M*. *carlylei*, with the Mussentuchit fragment BYU E-200 the holotype specimen.

### Geological setting

The Mussentuchit Member, which is the uppermost member of the more regionally widespread Cedar Mountain Formation, is exposed across central Utah, along the western side of the San Rafael Swell anticline east of the Wasatch and Fish Lake Plateaus, (Emery County, Utah). The Mussentuchit Member, the uppermost member of the Cedar Mountain Formation, spans an ~800,000 year interval within the earliest Cenomanian (99.674 + 0.439 / − 0.197 to 98.905 + 0.158 / − 0.183 Ma), with locally traceable, highly unaltered volcanic ash zones (MAZ1-MAZ4) providing spectacular temporal resolution [1]. Preserved sedimentary successions indicate that the Mussentuchit Member was a paralic depocenter (distal fluvial to coastal-tidal flat) margin along the western margin of the Interior Seaway that split North America during much of the Cretaceous [4, 37, 38].

## Methodology and materials

Institutional Abbreviations—BLM, Bureau of Land Management, Washington DC; BYU, Brigham Young University, Provo, Utah, U.S.A; FMNH, Field Museum, Chicago, Illinois, U.S.A.; NCSM, North Carolina Museum of Natural Sciences, Raleigh, North Carolina, U.S.A; NCSU, North Carolina State University, Raleigh, North Carolina; OMNH, Sam Noble Oklahoma Museum of Natural History, Norman, Oklahoma, U.S.A.; UGS, Utah Geological Survey, Salt Lake City, Utah.

Anatomical Abbreviations—CL, continuous layer (or crystalline layer); ML, mammillary layer; TST, total shell thickness.

### Materials

In this study, we examined all fossilized eggshells recovered from the Mussentuchit Member of the Cedar Mountain Formation and housed at the NCSM (n > 2,500), plus additional loaned material from the OMNH (n > 1,500), FMNH (n = 77), and BYU (n = 8), to extensively analyze ootaxonomic diversity across twenty localities. NCSM and FMNH fragments from the Mussentuchit Member were collected between 2012 and 2022, and fragments and thin section slides loaned from BYU and the OMNH were collected by Jensen [26] and Cifelli et al. [2], respectively.

### Methodology

We systematically categorized fragments of eggshell into ‘morphotypes’ based on external ornamentation patterns, and selected the highest quality samples (free of additional matrix, appropriately sized) for photography and consumptive sampling. Fragments selected were photographed with a Keyence VHX-7000 Digital Microscope before creating radial thin sections to assess microstructural features such as crystal arrangement, pore networks, the ML/CL boundary, and ML/CL thickness ratios. We encased fragments in epoxy resin, cut the resulting blocks using a Buehler IsoMet 1000 Precision Saw, and polished them using a Buehler MetaServ 250 Grinder/Polisher to a thickness of 80 μm. We analyzed finalized thin sections with a Nikon Eclipse Ci Pol light microscope (with attached DS-FI 2 camera) and the Keyence VHX-7000 Digital Microscope for microstructure. Measurements of TST, ML and CL features were taken digitally using ImageJ. Further analysis of microstructure and ultrastructure, with a focus on crystal splaying across the ML/CL boundary, was undertaken on a Hitachi SU8700 field-emission scanning electron microscope at the NCSU Analytical Instrumentation Facility. We viewed samples at 100x magnification at variable pressure, with a 50 Pa backfill of dry nitrogen and a 20 kV accelerating voltage. These combined analyses were used to determine ootaxonomic assignment.

### Ethics statement

Specimens collected for this study were obtained under the following permits from BLM (UT15-001, UT14-008E, UT15-003E, UT17-007E, UT20-006E, UT13-017E, 2021–584, UT-017E, UT15-001S) and UGS (2020–569, 2022–601). All necessary permits were obtained for the described study, which complied with all relevant regulations.

### Systematic paleontology

Oofamily ELONGATOOLITHIDAE Zhao 1975

Included Oogenera—*Elongatoolithus* Zhao and Jiang, 1974 (= Oolithes *elongatus* Young, 1954); *Macroolithus* Zhao, 1975 (= *Oolithes rugustus* Young, 1965); *Nanhsiungoolithus* Zhao, 1975 (= *Oolithes nanhsiungensis* Young, 1965); *Ellipsoolithus* Mohabey, 1998; *Trachoolithus* Mikhailov, 1994; *Macroelongatoolithus* Li, Yin, and Liu, 1995 (see Wang and Zhou [34], for oofamily Macroelongatoolithidae and Wang et al., 2010, for assignment of oogenus Macroelongatoolithus to oofamily Macroelongatoolithidae, and introduction of the oogenus *Megafusoolithus*); *Undulatoolithus* Wang, Zhao, Wang, Li, and Zou, 2013.

Referred Specimen—NCSM 33736 (n = 1), Cenomanian Mussentuchit Member of the Cedar Mountain Formation, Emery County, Utah, U.S.A.

Revised Diagnosis—Eggs are elongate and range from 9–61 cm long. TST 0.61–4.74 mm depending on oogenus and oospecies. CL and ML thickness ratio ranges from ca. 2:1–8:1. Eggshell with two distinct structural layers, a mammillary layer consisting of radiating calcite crystals and a second structural layer lacking well-defined shell units, obscured by squamatic ultrastructure, with undulating accretion lines. CL separated from the underlying ML by a distinct boundary. Pore systems are variable (angusticanaliculate most common). Dispersituberculate, ramotuberculate, and lineartuberculate ornamentation types are common.

Description—A single eggshell fragment from the ‘Kate’s Ridge’ locality. TST is 0.98–1.42 mm including ornamentation. Ratio of CL:ML is ca. 3:1. Eggshell in cross-section shows two distinct layers demarcated with a clear boundary; the lower ML has sub-vertical crystals extending from nucleation sites, and the overlying CL has no distinct shell units but exhibits clear wavy accretion lines. Pores appear to be prolatocanaliculate. External ornamentation is coarse and ramotuberculate (a combination of nodes and ridges with weak orientation), with heights ca. 1/5 of TST at the apex of ornamentation.

Remarks—We revise the diagnosis of Elongatoolithidae from Simon et al. [25] to assign all previously described *Continuoolithus* specimens to this oofamily. This is detailed in the *Continuoolithus canadensis* Systematic Paleontology section of this paper.

Previously, all elongatoolithid fragments from the Mussentuchit were assigned to *M*. *carlylei* [32], presumably on the assumption that only a single elongatoolithid ootype was present in the member. We advise caution in assigning fragments with this degree of granularity. There is little difference in the microstructural features across Elongatoolithidae, with the exception of the genus *Macroelongatoolithus*, which is diagnosable based on crystal splaying across the ML/CL boundary [25, 39], but this feature was not reported in Zelenitsky et al. [32]. Although commonly seen, not all Elongatoolithidae morphotypes from the Mussentuchit exhibit this feature. NCSM 33736 not only lacks crystal splaying across the ML/CL boundary, but lies outside the diagnosable TST range for the oogenus *Macroelongatoolithus*. The oogenus this fragment most resembles visually is *Ellipsoolithus*, known only from the Maastrichtian Lameta Formation of India [40]. However, we do not formally refer this specimen to the oogenus, as the initial description of *Ellipsoolithus* lacks microstructural characteristics in the description that would allow for confident referral of an isolated fragment.

Oofamily ELONGATOOLITHIDAE Zhao 1975

Oogenus *MACROELONGATOOLITHUS* Li, Yin, and Liu, 1995

Oospecies *MACROELONGATOOLITHUS CARLYLEI* Jensen, 1970

Junior Synonyms—*Oolithes carlylensis* Jensen, 1970:62–63, pl. 1, figs. 1, 2, 4, 6; pl. 2, figs. 3, 5, 6; pl. 3, figs. 4, 7, 8; text-fig. 5.; Macroelongatoolithidae Wang and Zhou, 1995 (see also Wang et al., 2010); *Boletuoolithus* Bray, 1998:221–222, figs. 1–3, 4a, b.; *Macroolithus carlylei* (Jensen, 1970); Zhao, 1975:108; *Longiteresoolithus xixiaensis* Wang and Zhou, 1995:262; Zhou et al. 1999:298–299, fig. 1b, d; Zhou et al., 2001:98; Liang et al., 2009:fig. 2h; *Macroelongatoolithus xixiaensis* Li et al., 1995:pl. 1.; *Macroelongatoolithus xixiaensis* Fang et al., 1998:pl. 17, fig. 10; Grellet-Tinner et al., 2006:figs. 6d–f, 7a–f; Wang et al., 2010:figs. 3a–d, 4a–d; Huh et al., 2014:figs. 3, 5a, 6a–d, 7, 8; *Macroelongatoolithus xixia* Carpenter, 1999:fig. AII.22. *Macroelongatoolithus sp*. Carpenter, 1999:fig. 10.12. *Macroelongatoolithus goseongensis* Kim et al., 2011b:figs. 2a, 5a–h; *Megafusoolithus qiaoxianensis* Wang, Zhao, Wang, Jiang, and Zhang, 2010b:fig. 5a–e.

Referred Specimens—Eggshell fragments NCSM 33724 (n = 3), NCSM 33725, NCSM 33726, NCSM 33734, NCSM 33735, NCSM 33737, NCSM 33756 (n = 9), NCSM 33813, NCSM 33815, NCSM 33816, NCSM 33817, NCSM 33818, NCSM 33820, NCSM 33822 (ntotal = 24), Cenomanian Mussentuchit Member of the Cedar Mountain Formation, Emery County, Utah, U.S.A.

Revised Diagnosis—Large, elongate eggs exceeding 250 mm and up to 610 mm long. TST, including ornamentation, is 1.38–4.75 mm. Abrupt, undulating boundary separates the CL and ML. CL:ML ratio ranges widely from 2:1 to 8:1. Crystal splaying across the ML/CL boundary occurs at random intervals. Ornamentation is variable both in type and relief and may grade from dispersituberculate at the poles to lineartuberculate along the middle portion of the length of the egg; smooth patches may occur near poles. Clutches consist of paired eggs in a ring-shaped configuration with an external clutch diameter of up to 3 m.

Distribution—Upper Cretaceous Zoumagang, Majiacun, Gaogou, Zhaoying, and Sigou formations, Henan Province, Xixia Basin, China; Albian—Cenomanian Liangtoutang Formation and Upper Cretaceous Chichengshan Formation, Zhejiang Province, Tiantai Basin, China; Upper Cretaceous Goseong Formation, South Gyeongsang Province, South Korea; Lower Cretaceous Cedar Mountain, Kelvin, and Naturita Formations, Utah, U.S.A.; Albian—Cenomanian Wayan Formation, Bonneville County, Idaho, U.S.A.; Cenomanian—Turonian Liangtoutang Formation, Zhejiang Province, Tiantai Basin, China [25].

**Fig 2:**
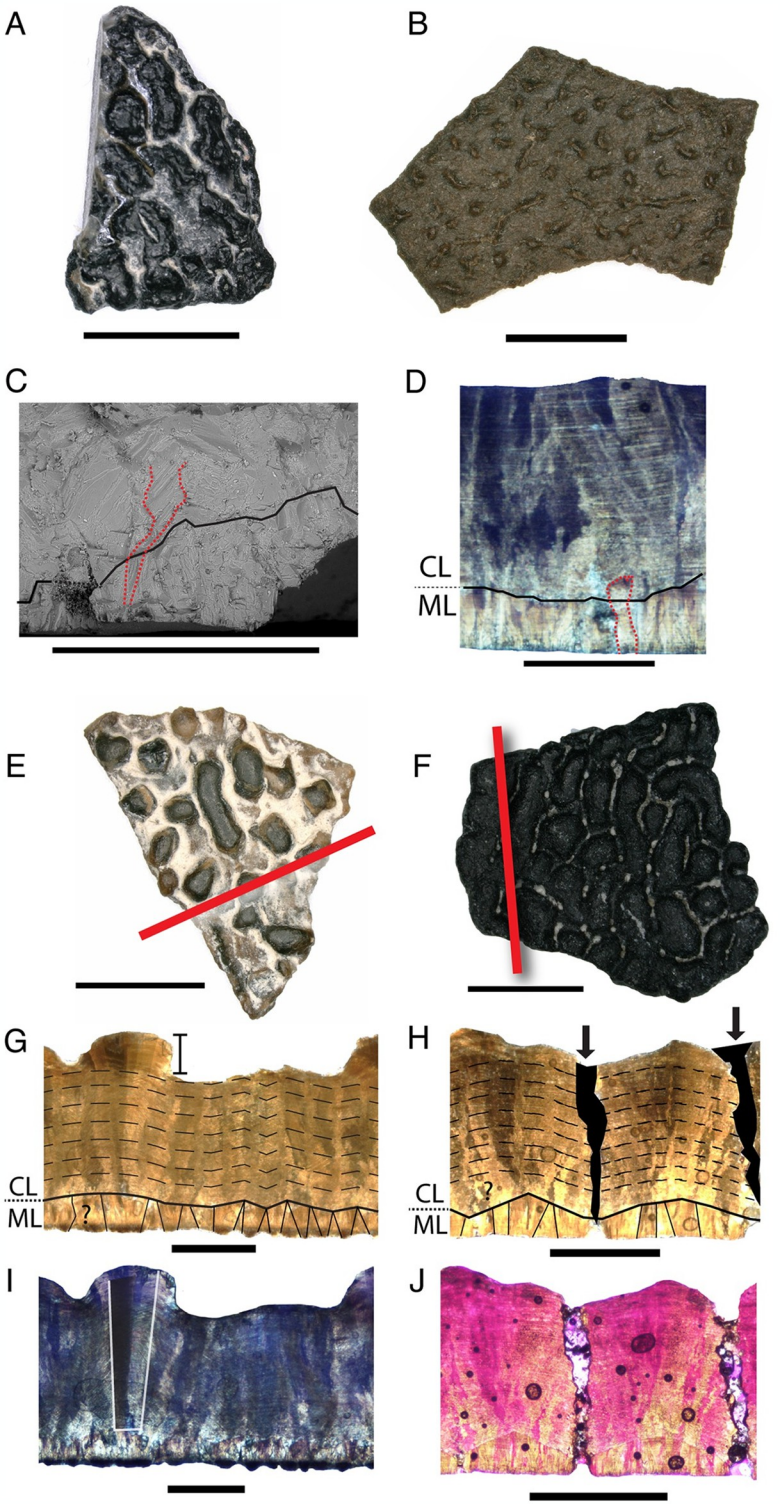
*Macroelongatoolithus* ‘morphotypes’ showing the variation in surface ornamentation patterns, and diagnostic features of microstructure. A) BYU E-200 (holotype) in surface view; B) NCSM 33734 in surface view. C–D Cross sections of NCSM 33725 showing crystal splaying (red dashed line) across the ML/CL boundary (solid black line) in C) scanning electron microscopy and D) cross-polarized light. E–J Fragments of NCSM 33576 [E), G), I)] and NCSM 33822 [F), H), J)] through the thin sectioning process, demonstrating variation in ornamentation and shared characters in thin section E) NCSM 33576 in surface view; F) NCSM 33822 in surface view; G) NCSM 33576 in radial cross section under plane polarized light, tailed scale bar shows extent of ornamentation, mammillae and shell units outlined in ML; H) NCSM 33822 in radial cross section under plane polarized light, arrows and black fill show infilled pores. I) NCSM 33576 in radial cross section under crossed polars with lambda filter, blocky extinction outlined in white. J) NCSM 33822 in radial cross section under crossed polars with lambda filter. Solid red lines indicate where thin sections were cut; solid black line indicates ML/CL boundary; dashed black lines indicate accretion lines across squamatic ultrastructure of the CL obscuring individual shell units. All scale bars = 10 mm.

Description—Eggshell fragments are associated with two clutches of eggs from the ‘Deep Eddy’ NCSM 33576) and ‘Holy Hand Grenade’ (NCSM 33724) field localities, as well as several other sites. External ornamentation is variable (Fig 2A and 2B), including high relief nodes and ridges, linear ridges with consistent orientation, to disperse nodes and very low relief to near-smooth surfaces.

In radial thin section, TST ranges from 1.38–2.33 mm. Eggshells are composed of two layers delineated by a sharp, undulating boundary (Fig 2). The CL:ML ratio ranges between 3:1 and 8.5:1. Ornamentation can be up to 1/3 of eggshell thickness (Fig 2G). Pores are a range of straight and narrow (‘angusticanliculate’), angled and straight (‘oblicucanaliculate’), or variable in width (‘prolatocanaliculate’; Fig 2H). The eggshell units are obscured in the CL by squamatic ultrastructure. Under cross-polarized light, extinction patterns are columnar or blocky (Fig 2I). Eggs under scanning electron microscopy and cross-polarized light show crystal splaying across the ML/CL boundary at random intervals (Fig 2C and 2D).

Remarks—The discovery of whole eggs at the ‘Deep Eddy’ and ‘Holy Hand Grenade’ localities requires amendment to the diagnosis provided by Simon et al. [25]. Whole eggs from these two localities fall below the lower threshold for the pre-existing *Macroelongatoolithus carlylei* diagnosis of 34 cm egg length (‘Deep Eddy’ eggs range from 27.1–30.1 cm; ‘Holy Hand Grenade’ from 25.0–25.9 cm). Therefore, we have amended the oospecific diagnosis to expand the lower bound for egg length from 34 to 25 cm. Eggs in the nest have been crushed and telescoped, but we estimate variation in the lengths has not been altered by more than 1 cm in either direction.

Specimens now referred to *Macroelongatoolithus* have a convoluted diagnostic and descriptive history. In the 1960s, fragments from the Mussentuchit Member were described as *Oolithes carlylensis* [26] and with the advent of egg parataxonomy, were assigned to Elongatoolithidae [29]. From the 1990s, extensive finds in Asia dramatically expanded the diversity of *Macroelongatoolithus* and other colossal oviraptorosaur eggs, resulting in the naming of three oogenera and five oospecies of Elongatoolithidae [34, 36, 37]. Zelenitsky et al. [32] proposed that all known ootaxa had significant microstructural overlap and were junior synonyms of the original material from the Mussentuchit Member. They amended the Jensen [26] nomenclature and erected *Macroelongatoolithus carlylei* to encompass all of these taxa. This single oospecies identification is inconsistently applied, with ootaxa from Asia described before and since still referred to other oospecies (*M*. *xixiaensis*, *M*. *goseongensis*), or the oofamily Macroelongatoolithidae. Simon et al. [25] described the first whole egg pair of *M*. *carlylei* in North America from the Wayan Formation of Idaho. Overlap in microstructural features in this paper supports the Zelenitsky et al. [32] justification of referring all *Macroelongatoolithus* species, plus *Megafusoolithus* and *Longiteresoolithus* to *M*. *carlylei*. The diagnosis provided by Simon et al. [25] permits us to assign these eggshells to *M*. *carlylei* based on the presence of crystal splaying across the ML/CL boundary, which occurs only in this ootaxon within Elongatoolithidae [25] and has been suggested as a method of strengthening the eggshell with increased size [39].

Oogenus *UNDULATOOLITHUS* Wang, Zhao, Wang, Li and Zhou 2013

Oospecies *UNDULATOOLITHUS PENGI* Wang, Zhao, Wang, Li and Zhou 2013

Referred Specimens—Eggshell fragment NCSM 33729 (n = 1), Cenomanian Mussentuchit Member of the Cedar Mountain Formation, Emery County, Utah, U.S.A.

Revised Diagnosis—TST ranges from 0.78–2.16 mm (including ornamentation). The outer surface of the eggshell is ornamented with nodes and longitudinally aligned ridges, and the ridges are ca. 1/2 of the TST. CL:ML ratio ranges from 4:1–8:1, with a gradational boundary between layers.

Distribution—Cenomanian Mussentuchit Member, Cedar Mountain Formation, Utah, U.S.A.; Upper Cretaceous Pingxiang Basin, Jiangxi Province, China.

**Fig 3:**
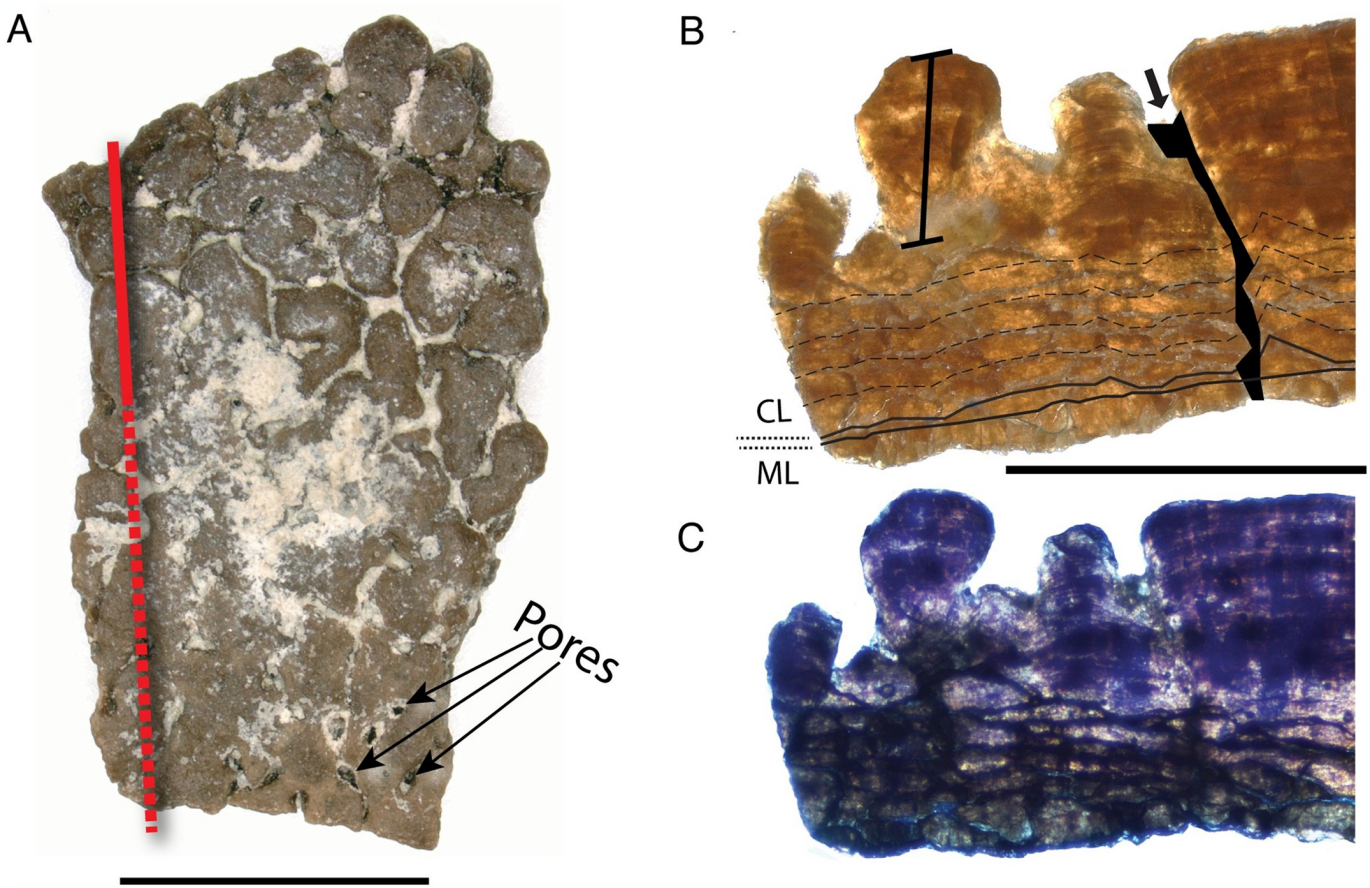
Features of *Undulatoolithus* fragment NCSM 33729. A) surface view of NCSM 33729. Red line indicates where thin section was made (dashed where not seen in the radial thin sections). B) NCSM 33729 in radial thin section. Tailed scale bar marks very high, undulating ornamentation. Gradational ML/CL boundary marked with solid black lines; accretion lines in CL shown with dashed black lines; arrows and black fill show infilled pores. C) radial thin section under crossed polars. Scale bars equal 10 mm.

Description—The eggshell surface is composed of rugose nodes intermittently coalescing into ridges (Fig 3A), and significant mineral overgrowth. Pores appear slightly elongated and are low-density.

In radial thin section, two eggshell layers have an unclear boundary obscured by cracks (Fig 3B). CL:ML ratio ranges from 4.6:1–5.8:1. TST ranges from 1.17–2.16 mm (with ornamentation). Ornamentation is extreme, constituting nearly one half of TST, and can be as tall as 0.98 mm. Complete pores are not visible in a single cross-section but appear to be straight and narrow (angusticanaliculate). Accretion lines are visible across eggshell units in the continuous layer, with individual units obscured by squamatic ultrastructure. In cross-polarized light, extinction patterns are blocky where not obscured (Fig 3C).

Remarks—*Undulatoolithus pengi* was previously known from a single locality in Jiangxi Province, China [41] that included whole eggs arranged in a partial nest. We revise their diagnosis based on a greater shell thickness ratio in the Mussentuchit Member fragments, and remove the character of egg size from the diagnosis due to the significant overlap with other elongatoolithid oogenera. The described specimen from the Mussentuchit Member has many of the characteristics of the Asian eggs of *U*. *pengi*, most notably the remarkable ornamentation-to-shell thickness ratio, and conforms with the microstructural diagnosis. The presence of the remarkable ornamentation ratio encompassing up to 50% of TST excludes it from being referred to other elongatoolithid taxa. The identification of *Undulatoolithus* in the Mussentuchit Member of the Cedar Mountain Formation extends the spatiotemporal range of the oogenus to the early Late Cretaceous of North America from the Late Cretaceous of China.

Oofamily ELONGATOOLITHIDAE Zhao, 1975

Oogenus *CONTINUOOLITHUS* Zelenitsky, Hills, and Currie 1996

Oospecies *CONTINUOOLITHUS CANADENSIS* Zelenitsky, Hills, and Currie, 1996

Junior Synonyms—*Spongioolithus hirschi* Bray 1999 pp. 368–369, fig. 5

Type Oospecies—*Continuoolithus canadensis* Zelenitsky, Hills and Currie 1996

Referred Specimens—NCSM 33727, NCSM 33728, NCSM 33732, NCSM 33733, NCSM 33738 (n = 5), Cenomanian Mussentuchit Member, Cedar Mountain Formation, Utah, U.S.A.

Revised Diagnosis—Eggs are approximately 120 mm long x 55 mm wide, elongated, and occur in pairs. External surface ornamented primarily by isolated and coalesced nodes, with occasional ridges. TST ranges from 0.61–1.74 mm with ornamentation. Pores are usually located between or at the base of nodes or ridges, and the pore system is ‘angusticanaliculate’. Two structural layers are present, with a ML:CL ratio of ca. 1:6–1:8. Adjacent mamillae tightly abutting one another.

Distribution—Lower Maastrichtian Willow Creek Formation, Alberta, Canada; Maastrichtian North Horn Formation, Utah, U.S.A.; Maastrichtian St. Mary River Formation, Alberta, Canada.; Campanian Two Medicine Formation, Montana, U.S.A.; Campanian Kaiparowits Formation, Utah, U.S.A.; Campanian Dinosaur Park Formation, Alberta, Canada; Campanian Oldman Formation, Alberta, Canada; Campanian Fruitland Formation, New Mexico, U.S.A.; Campanian Aguja Formation, Texas, U.S.A.; Campanian El Gallo Formation, Spain; Santonian Milk River Formation, Alberta, Canada; Cenomanian Mussentuchit Member, Cedar Mountain Formation, Utah, U.S.A.; Morrison Formation, New Mexico, U.S.A.

**Fig 4:**
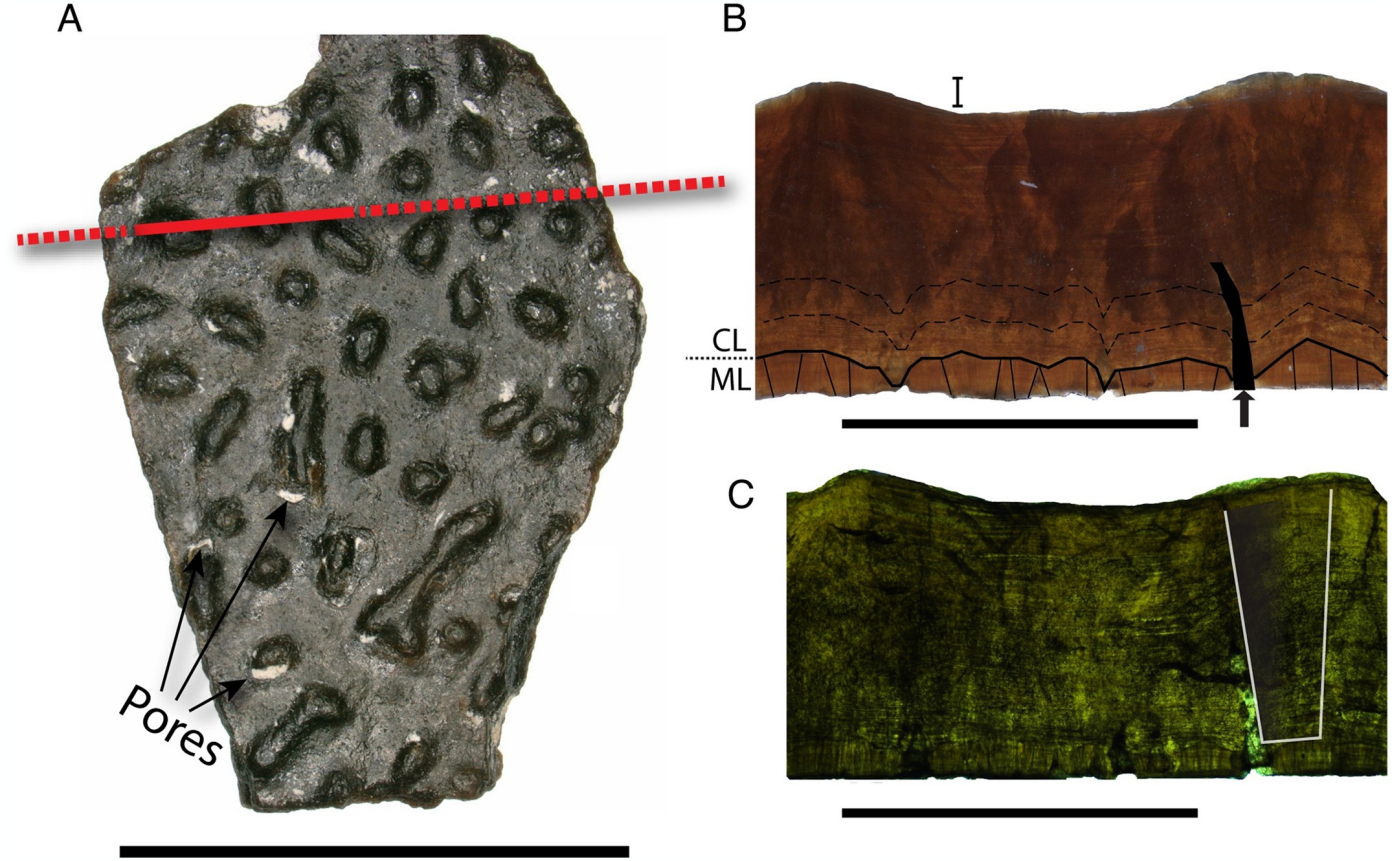
Features of *Continuoolithus* fragments. A) NCSM 33728 in surface view, showing characteristic nodes with intermittent coalescence. Red line indicates where thin section was made (dashed where not seen in the radial thin sections). B) NCSM 33728 in radial thin section. ML/CL boundary highlighted with solid black line; mamillae and shell units outlined in ML; pore infilled in black and marked with arrow; dashed black lines indicate accretion lines in CL. C) NCSM 33728 in radial thin section with crossed polars. White outline shows a blocky extinction pattern within shell units. All scale bars equal 10 mm.

Description—Fragments have nodose ornamentation with some nodes coalescing into ridges. Pore openings are rounded to elongate and usually occur adjacent to nodes (Fig 4A).

In radial thin section, the CL and ML are delineated with a clear, undulating boundary, with a thickness ratio ranging from 6:1–8:1. TST ranges from 1.01–1.74 mm. Ornamentation varies between 1/7 to 1/5 of TST. Pores are straight and narrow (‘angusticanaliculate’; Fig 4B). Accretion lines are present and undulating in the CL, but individual eggshell units are not seen in the continuous layer. Extinction patterns in cross-polarized light are columnar (Fig 4C).

Remarks—We revise the diagnosis of *Continuoolithus canadensis* from [42] to include trivial increases to the upper limit of eggshell thickness found in this study (from 1.73–1.74 mm) and ML:CL ratios (from ca. 6.5–7:1 in Voris et al. [42], to 6:1–8:1 in this study).

Since its formal diagnosis [43], *Continuoolithus* has been recognized as similar to Elongatoolithidae eggs, but has only once been formally included in the oofamily [36], instead being most commonly informally assigned to Theropoda [33, 43]. The key differentiator has been the particular external ornamentation style, with isolated nodes that sometimes coalesce, and lower total shell thickness [33, 44]. Although diagnostic skeletal material would be preferable, we assign *Continuoolithus* to Elongatoolithidae based on the following suite of elongatoolithid characters from the diagnosis here that conform to the diagnosis of Elongatoolithidae in Simon et al. [25] and this study: elongated eggs laid in pairs between 9–61 cm in length, and a shape index < 50 [45]; CL and ML thickness ratio ranging from ca. 6:1–8:1; eggshell with two distinct structural layers, a mammillary layer consisting of radiating calcite crystals and a second structural layer lacking well-defined shell units with undulating accretion lines separated from the underlying ML by a distinct boundary; dispersituberculate ornamentation type with coalescing nodes. We have five morphotypes in this study that also have a shell thickness well within the known Elongatoolithidae range (1.01–1.74 mm), but we extend the lower bound of Elongatoolithidae thickness from 0.8 to 0.61 mm given the suite of other features that are concomitant with the oofamily. We do note however that previous measurements taken with fragments may lower TST values if not considering the resorption of mammillae nucleation sites at the base of the ML due to embryo developmental differences.

Oofamily SPHEROOLITHIDAE Zhao 1979 emend. Mikhailov 1991

Oogenus *SPHEROOLITHUS* Zhao 1979 emend. Mikhailov 1994

Oospecies *SPHEROOLITHUS ALBERTENSIS* Zelenitsky and Hills 1997

Referred Specimens—NCSM 33731 (n = 1), NCSM 33814 (n = 1), Cenomanian Mussentuchit Member of the Cedar Mountain Formation, Emery County, Utah, U.S.A.

Diagnosis—Sub-spherical eggs with spheroolithid microstructure, TST of 0.98–1.22 mm, primarily fine sagenotuberculate ornamentation, dominantly prolatocanaliculate with some rimocanaliculate pores [42].

Distribution—Maastrichtian North Horn Formation, Utah, U.S.A; Maastrichtian Willow Creek Formation, Alberta, Canada; Campanian Oldman Formation, Alberta, Canada; Campanian Dinosaur Park Formation, Alberta, Canada; Campanian Two Medicine Formation, Montana, U.S.A.; Campanian Kaiparowits Formation, Utah, U.S.A; Santonian Milk River Formation, Alberta, Canada; Cenomanian Mussentuchit Member, Cedar Mountain Formation, Utah, U.S.A..

**Fig 5:**
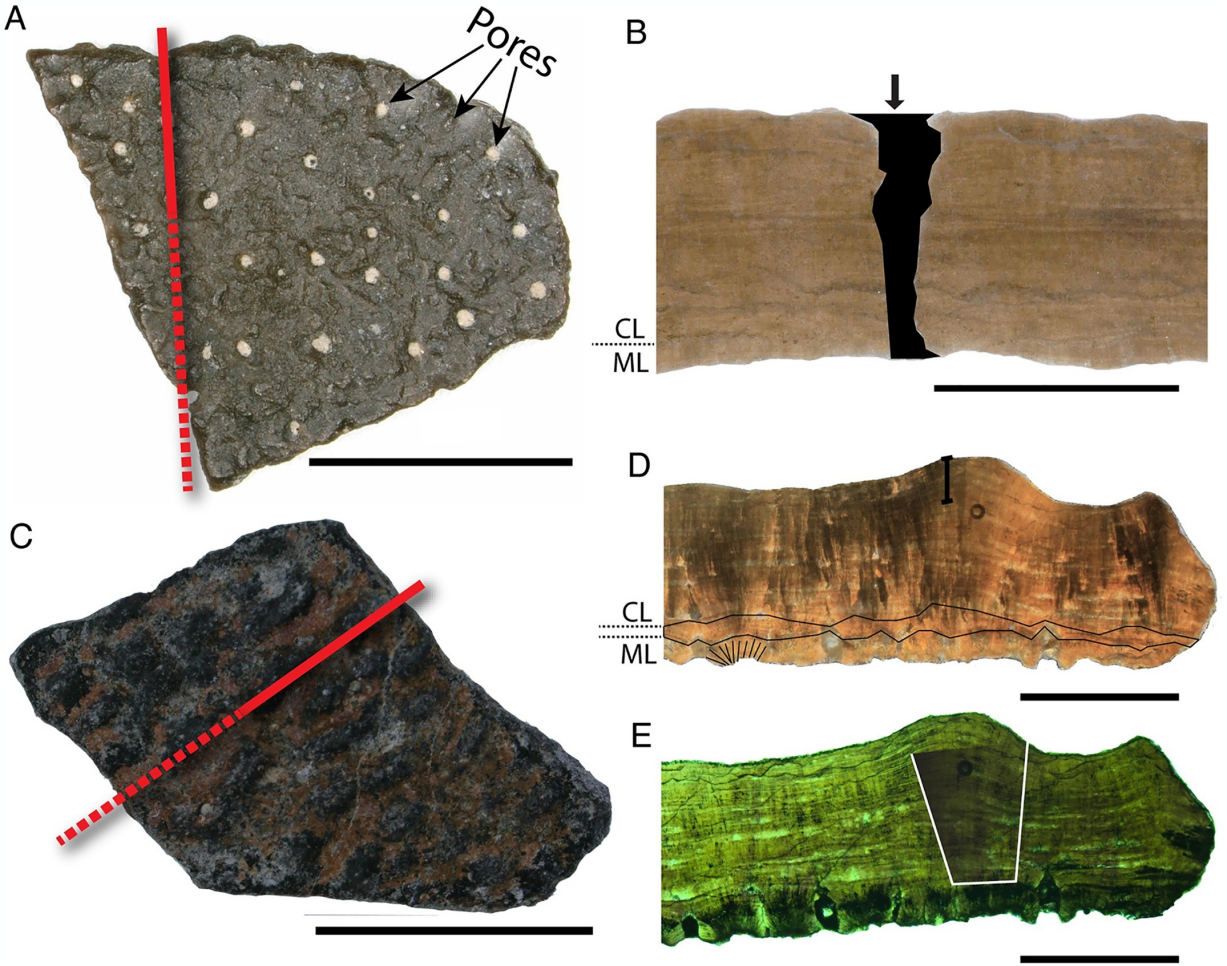
Features of *Spheroolithus* fragments. A–B *S*. *albertensis*. A) NCSM 33731, in surface view showing low anastomosing ridges and large spherical pores, scale bar equals 5mm. B) NCSM 33731 in radial thin section with funnel-shaped pore infilled in black and marked with arrow. C–E *S*. *cf*. *europaeus*. C) OMNH 28280 in surface view with rugose linear ridges. D) OMNH 28280 in radial thin section, CL/ML boundary is fluctuant (solid black lines) and individual shell units with radiating spherulites in the ML are identifiable. E) OMNH 28280 in radial thin section with crossed polars. White outline shows a sweeping extinction pattern within shell units. All scale bars equal 10 mm.

Description—Eggshell external surfaces have low-relief ornamentation composed primarily of fine, anastomosing ridges (Fig 5A). Pore openings are sub-circular, and particularly dense in NCSM 33731. In radial thin section, the eggshell is not clearly split into two distinct layers but an irregular boundary is present where shell units become fused (Fig 5B). TST ranges from 1.01–1.11 mm. The ratio between the upper and lower layers is approximately 4:1. Eggshell units, although fused, are distinguishable by their sweeping extinction patterns in cross-polarized light and individual mammillae with radiating crystals. Pores, where seen, are funnel-shaped and taper inwards (‘rimocanaliculate’, Fig 5B). Ornamentation is thin and represents between 1/13 and 1/8 of TST.

Remarks—*Spheroolithus* is a multi-oospecific oogenus within the oofamily Spheroolithidae [46], although see Stromatoolithidae in Zhu et al. [47]. It has significant northern hemisphere distribution in North America, Europe and Asia. We assign NCSM 33731 and NCSM 33814 to *Spheroolithus albertensis* based on the following suite of characters: eggshell thickness ranging from 1.01–1.11 mm; sweeping extinction in cross-polarized light, distinct eggshell units unobscured by squamatic ultrastructure; ornamentation composed of thin, anastomosing ridges; high pore density of sub-circular aperture, and rimocanaliculate pores [43]. We distinguish it from *S*. *irenensis* [48] and *S*. *tenuicorticus* [49] by the presence of anastomosing ridges of ornamentation, and the thickness of the eggshell (1.08 mm) differentiates our eggshell from *S*. *chiangchiungtingensis* (2.7 mm; [48]), *S*. *megadermus* (5.7 mm [27]), and *S*. *choteauensis* (0.66–0.94 mm [50]). It also lacks the densely concentrated, undulating accretion lines in the radial microstructure of *S*. *europaeus* [51].

Referral of specimens in the Mussentuchit Member to *S*. *albertensis* extends the range of this oospecies, and the *Spheroolithus* oogenus in the Western Interior Basin, from the Santonian of the Milk River Formation (ca. 84.5–83.5 Ma; [52]) back into the Cenomanian by ca. 15 Myr. Although this referral represents the first description of *Spheroolithus* from the Mussentuchit Member, it does not represent the first argument for Spheroolithidae eggshell in the member. Several decades ago Bray [31] erected *Boletuoolithus* within Spheroolithidae for a sample of eggshell fragments collected from the Mussentuchit Member. Zelenitsky et al. [32] examined these fragments, concluded that these specimens were misidentified, and referred them to *M*. *carlylei*, removing Spheroolithidae from the recognized paleooodiversity of the Mussentuchit Member. We agree with Zelenitsky et al. [32] that the fragments described by Bray [31] are not referrable to Spheroolithidae. However, we now independently document the presence of spheroolithid eggshells from the member based on newly collected materials that are referable to *Spheroolithus albertensis*.

*Spheroolithus albertensis* is widely distributed across the Late Cretaceous Western Interior Basin, including whole eggs in the Lower Two Medicine Formation [50], plus fragments from the Oldman Formation [53], St Mary River Formation [43, 54], the Kaiparowits Formation [55], and the Milk River Formation [52]. Shell thickness in the Mussentuchit Member spheroolithid eggshell (1.08 mm) is on average closest to eggshell from the St Mary River Formation (1.08 mm [42], but with a significantly lower range of thicknesses (1.01–1.11 mm, n = 2), owing most likely to a lower number of samples than other later Cretaceous assemblages (Table 1).

**Table 1:** Known oodiversity of the Cretaceous Western Interior Basin of North America. The Mussentuchit Member oogenera are shown in bold.

Oogenus	Formation	Age	Location	Reference
*Continuoolithus*, *Montanoolithus*, *Porituberoolithus*, *Prismatoolithus*	Willow Creek	Upper Maastrichtian	Alberta	Zelenitsky et al. (2017a)
*Continuoolithus*, *Ovaloolithus (two species)*, *Spheruprismatoolithus*	North Horn	Upper Maastrichtian	Utah	Bray (1999)
*Belonoolithus*, *Dimorphoolithus*, *Krokolithes*, *Spheroolithus*, *Testudoolithus*,	Hell Creek	Maastrichtian	Montana	Jackson and Varricchio (2016)
*Continuoolithus*, *Montanoolithus*, *Prismatoolithus*, *Tetonoolithus*, *Spheroolithus*	St. Mary River	Lower Maastrichtian	Alberta, Montana	Voris et al., (2018); Jackson and Varricchio ((2017)
*Continuoolithus*, *Montanoolithus*, *Prismatoolithus*	Upper Two Medicine	Upper Campanian	Montana	Horner and Makela (1979); Horner (1982); Hirsch and Quinn (1990); Horner and Currie (1994); Zelenitsky and Hills (1996); Zelenitsky et al. (1996); Varricchio et al. (2002); Grellet-Tinner and Makovicky (2006); Zelenitsky and Therrien (2008a)
*Continuoolithus*, *Krokolithidae indet*., *Macroelongatoolithus*, *Prismatoolithus*, *Portituberoolithus*, *Spheroolithus*, *Stillatuberoolithus*	Kaiparowits	Upper Campanian	Utah	Oser et al. (2021)
*Continuoolithus*, *Porituberoolithus*, *Prismatoolithus*, *Reticuloolithus*, *Spheroolithus*	Dinosaur Park	Upper Campanian	Alberta	Zelenitsky and Sloboda (2005)
*Continuoolithus*, *Dispersituberoolithus*, *Porituberoolithus*, *Prismatoolithus*, *Tristaguloolithus*	Oldman	Upper Campanian	Alberta	Zelenitsky and Hills (1996); Zelenitsky et al. (1996); Zelenitsky and Hills (1997);
*Spheruprismatoolithus*, *Prismatoolithus*	Judith River	Upper Campanian	Montana	Zelenitsky and Hills (1996); Clouse (2001); Bray (1999); Jackson et al. (2010)
*Continuoolithus*, *Porituberoolithus*, *Prismatoolithus*	Fruitland	Upper Campanian	New Mexico	Tanaka et al. (2011)
*Continuoolithus*, *Porituberoolithus*	Aguja	Upper Campanian	Texas	Welsh and Sankey (2008)
*Prismatoolithus*, *Spheroolithus*, *Triprismatoolithus*, *Tubercuoolithus*, *Spheruprismatoolithus*	Lower Two Medicine	Lower Campanian	Montana	Bray (1999); Jackson and Varricchio (2010)
*Continuoolithus*, *Porituberoolithus*, *Prismatoolithus*, *Triprismatoolithus*	Milk River	Upper Santonian	Alberta	Zelenitsky et al. (2017b)
***Macroelongatoolithus*, *Undulatoolithus*, *Continuoolithus*, *Spheroolithus*, *Mycomorphoolithus***	**Cedar Mountain (Mussentuchit Member)**	**Cenomanian**	**Utah**	**Jensen (1970); Zelenitsky et al. (2000); This study**
*Macroelongatoolithus*	Wayan	Albian- Cenomanian	Idaho	Krumenacker et al. (2017); Simon et al. (2019)
*"D*. *antirrhopus" egg*	Cloverly	Aptian	Wyoming	Grellet-Tinner and Makovicky (2006)

Oofamily SPHEROOLITHIDAE Zhao 1979 emend. Mikhailov 1991

Oogenus *SPHEROOLITHUS* Zhao 1979 emend. Mikhailov 1994

Oospecies *SPHEROOLITHUS EUROPAEUS* Selles, Via and Galobart 2014

Referred Specimens—OMNH 28280 (n = 1), Cenomanian Mussentuchit Member of the Cedar Mountain Formation, Emery County, Utah, U.S.A.

Diagnosis—shell thickness ranging from 1.04 to 1.11 mm; sagenotuberculate surface dominated by fine irregular ridges; two types of pore apertures, large elliptical-shaped (300–600 *μ*m in width) and small rounded ones (100 *μ*m in diameter), sometimes paired; highly fused shell units; and undulating growth lines densely concentrated in the outer half of the shell [52].

Distribution—Maastrichtian Tremp Formation, Spain; Cenomanian Mussentuchit Member, Cedar Mountain Formation, Utah, U.S.A.

Description—Eggshell external surfaces have low-relief ornamentation composed primarily of irregular linear ridges. Pore openings are either small and sub-circular or large and ellipsoidal, but many are obscured by matrix or crystal overgrowth. In radial thin section, TST is ca. 1.08 mm. The eggshell is not clearly split into two distinct layers but an irregular gradational boundary is present where shell units become fused (Fig 5D). Pores are prolatocanalicuate. Eggshell units, although fused, are distinguishable by their sweeping extinction patterns in cross-polarized light and individual mammillae with radiating crystals (Fig 5E). In the thinnest eggshells there is erosion of the innermost shell either embryonically or taphonomically. Densely concentrated, undulating accretion lines are visible throughout the cross-section.

Remarks—OMNH 28280 has a different ornamentation pattern to *S*. *albertensis* specimens NCSM 33731 and NCSM 33814, with more linear, ‘worm-like’ ridges as reported in Sellés et al. [51]. This fragment has a sweeping extinction patterns, unclear ML:CL boundary, external ornamentation consistent with *Spheroolithus*. We refer it to *Spheroolithus europaeus* based on the presence of the following characteristics, consistent with the diagnosis from Sellés et al. [51]: shell thickness of 1.08 mm; external surface dominated by irregular linear ridges; large ellipsoidal and small sub-circular pores on the surface dense, undulating accretion lines through the continuous layer in radial cross section, and highly fused shell units. Our description extends the spatiotemporal range of *S*. *europaeus* from Maastrichtian Europe to Cenomanian North America.

INCERTAE SEDIS

Oogenus *MYCOMORPHOOLITHUS* Moreno-Azanza et al. 2015

Oospecies *MYCOMORPHOOLITHUS KOHRINGI* Moreno-Azanza et al. 2015

Referred Specimens—Eggshell fragments NCSM 35004, NCSM 35005 (n = 2), Cenomanian Mussentuchit Member of the Cedar Mountain Formation, Emery County, Utah, U.S.A.

Diagnosis—Eggshells characterized by mushroom-shaped or inverted subtriangular shell units comprising radiating wide crystals. TST ranges from 0.31–0.81 mm. Shell units are slender at the base of the unit, abruptly increasing up to fivefold in width at 1/3 to 1/2 the eggshell height. Pore openings are dense, vary in shape and dimensions, and may coalesce. Growth lines are straight to slightly wavy, over the entire eggshell thickness [56, 57].

Distribution—Cenomanian Mussentuchit Member, Cedar Mountain Formation, Utah, U.S.A.; Barremian Blesa Formation, Spain; Barremian El Castellar Formation, Spain; Barremian Mirambel Formation, Spain; Barremian La Huerguina Formation, Spain; Barremian El Collado Formation, Spain; Berriasian Purbeck Limestone Group, England.

**Fig 6:**
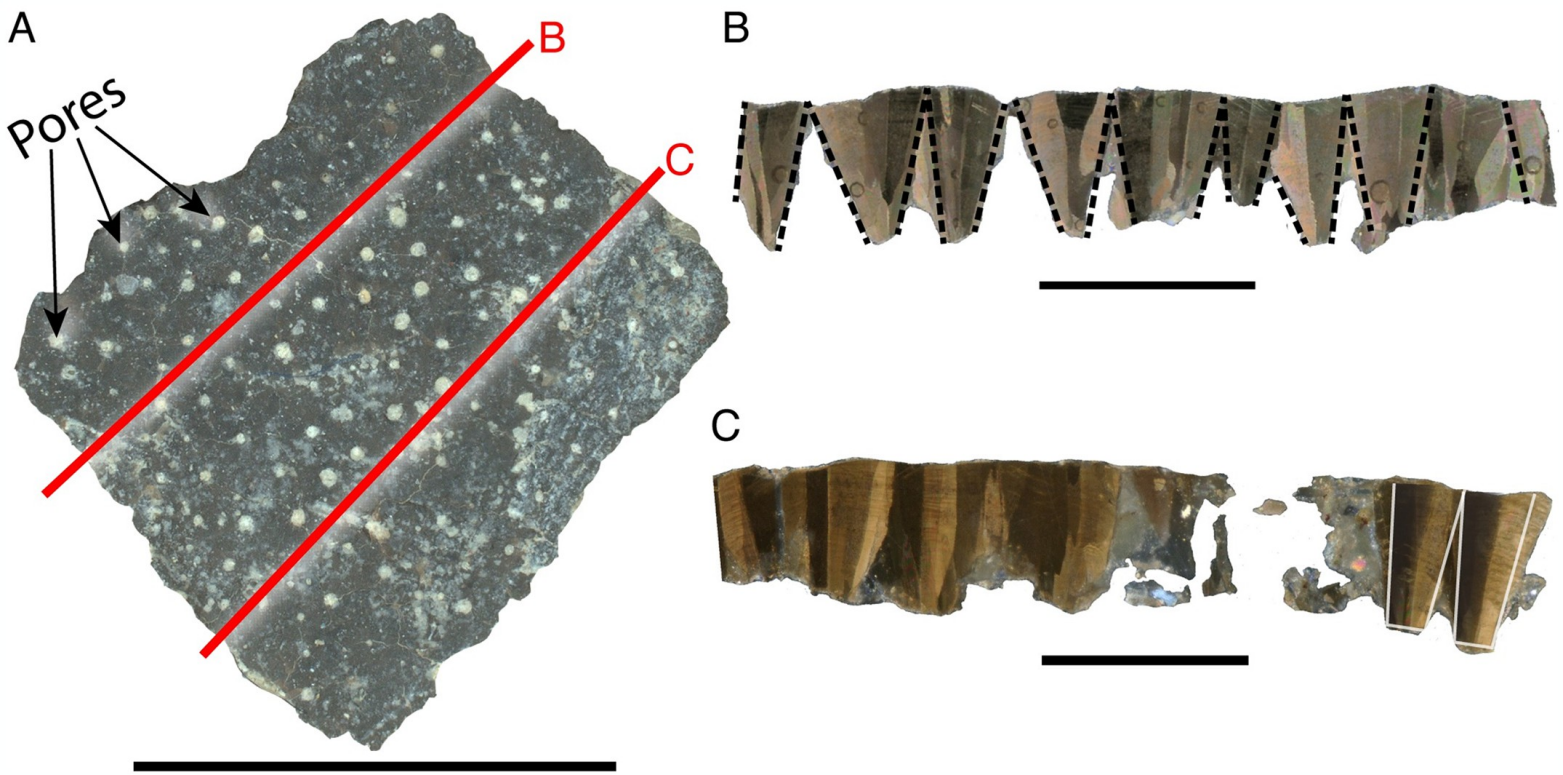
Features of *Mycomorphoolithus* fragment NCSM 35005. A) Surface view of NCSM 35005. Red lines indicate where thin sections were made. B-C NCSM 35005 in radial thin section showing single-layered triangular shell units (B), columnar extinction (white outlines) and dissolution of eggshell associated with crocodylomorph hatching (C).

Description—Eggshell surfaces lack ornamentation, with a high density of subcircular, relatively large (100–250μm) pore openings (Fig 6A). TST ranges from 0.64–0.72 mm. In radial thin section, eggshell is composed of a single layer for which individual eggshell units are sub-triangular to ‘mushroom-shaped’ [56], with the apices directed towards the egg interior (Fig 6B). Between units, eggshell exhibits large interstices, which obscure the nature of the pore system (Fig 6C). Under cross-polarized light, the eggshell displays blocky extinction within each eggshell unit.

Remarks—Currently, this ootaxon is restricted to a single oospecies, *M*. *kohringi* from Europe and is not formally assigned to any oofamily [56–59]. However, it is recognized as being most similar to eggs in the oofamily Krokolithidae, laid by crocodylomorph archosaurs [60, 61]. Whether these eggs can be assigned to Krokolithidae is uncertain because they lack tabular ultrastructure and organic basal cores that are characteristic of this oofamily [56], although see Bravo et al. [62]. Moreover, Moreno-Azanza et al. [63] suggest krokolithid eggshell is three-layered, not single layered as previously proposed, and that *Mycomorphoolithus* is single layered. Although Mycomorphoolithus is most likely crocodylomorph eggshell, there is currently no current justification for including it in Krokolithidae.

The crystal dissolution exhibited by extant crocodylomorphs [64, 65] is apparent in fossilized eggs, evidenced by a wide variety of pore aperture sizes and shapes exhibited by the Mussentuchit Member and European specimens [56]. This leads to a negative preservational bias and challenges studying microstructure, especially given the comparatively thin eggshell when compared to dinosaurian eggs (at maximum 0.81 mm). Eggshell fragments from the Mussentuchit Member fall within the full range of thickness in the existing diagnosis (0.31–0.81 mm), but mean thickness of the Mussentuchit Member specimens is higher than those from Europe (0.675 mm against 0.524 mm).

## Discussion

### Ootaxonomic affiliation

Assigning precise affinities between ootaxa and true taxa is speculative when there are no direct associations preserved. Nonetheless, assigning true taxonomic affinities at the highest granularity possible is important for studying faunistics and biogeography, and we therefore use the existing taxonomic data from the Mussentuchit Member, and the localities where the named ootaxa are also present, to make inferences.

All theropod eggshells described herein exhibit the diagnostic criteria for assignment to Elongatoolithidae. Elongatoolithid ootaxa present many body fossil associations with Oviraptorosauria and, to date, no other theropod clades [66–81]. However, the exclusive relationship between Oviraptorosauria and Elongatoolithidae merits further discussion due to studies noting similar morphological traits between eggshell associated with oviraptorosaurs and those associated with dromaeosaurs [82, 83] and phylogenetic studies documenting a close relationship between eggs attributable to dromaeosaurs and oviraptorosaurs [82–85]. These phylogenetic analyses often describe character traits with inconsistent or incomplete ootaxonomic nomenclature. Oviraptorosaurs and dromaeosaurs do share a symplesiomorphic suite of traits, along with the prismatoolithid eggs of Troodontidae,: paired eggs resulting from monoautochronic ovulation, two eggshell layers, dominantly angusticanaliculate pores, and eggs that are more elongate than spherical. Although these features are likely synapomorphic for Pennaraptora, they do not permit differentiation of oviraptorosaurian and dromaeosaurid eggs. Moreover, new research has shown that the previously recovered polyphyly of dromaeosaur and oviraptorosaur eggs may in fact be the result of erroneous assignment of some oogenera (*Heishanoolithus*, *Paraelongatoolithus*, possibly *Nanhsiungoolithus*) to Elongatoolithidae, which are more referable to ootaxa associated with dromaeosaurs [85, 86]. Putative dromaeosaur eggs, currently without a referable oofamily, have the following autapomorphies that distinguish them from oviraptorosaur eggs: less elongate eggs (Shape Index > 50 [85]), lower eggshell thickness ranging from 0.35–0.95 mm, loosely arranged and acicular mammillae in the ML, horizontal accretion lines in the CL, and reticulate ornamentation patterns [37, 43, 44, 85, 87–91], as well as unconfirmed sub-layering of the dromaeosaur egg continuous layer [83]. Although this does not change our taxonomic interpretation of the Mussentuchit Member ootaxa, we do recognize that relationships between eggs of dromaeosaurs and oviraptorosaurs remain poorly resolved phylogenetically. More data on dromaeosaur taxon-ootaxon relationships and a clear diagnosis created for dromaeosaur ootaxa that distinguishes these from Elongatoolithidae are required to resolve these issues.

Based on the number of elongatoolithid oospecies identified we can conclude that there were multiple oviraptorosaur species in the Mussentuchit Member. As the largest known oogenus within Elongatoolithidae, *Macroelongatoolithus* is commensurately attributed to the largest known oviraptorosaurs. This is corroborated by direct association between an egg and the perinate remains of *Beibeilong sinensis* [78], with size extrapolation suggesting a skeletally mature individual on the scale of *Gigantoraptor erlianensis* [92], the holotype of which is ca. 8 m in length and is estimated to have weighed over a tonne [93, 94]. The eggs associated with *Beibeilong* are 40–45cm in length [78], still well below the largest that have been reported (> 60 cm [35, 95–97]). These largest eggs stem from a nest that has considerable variation in egg length, up to 179% from shortest to longest (34–61 cm, [25]). Egg lengths for undescribed whole eggs from the Mussentuchit [98] show a range of 26–30 cm, outside the previous diagnosis for *Macroelongatoolithus* but certainly attributable to a giant oviraptorosaur on the order of magnitude for *Beibeilong* and *Gigantoraptor*. Giant oviraptorosaur body fossils have been excavated from the Mussentuchit Member of the Cedar Mountain Formation but remain undescribed [99, 100].

Whole egg remains or fragments of *Undulatoolithus* have yet to be found in association with any body fossils. Still, as *Undulatoolithus* is assigned to Elongatoolithidae, it most likely represents a different oviraptorosaur taxon. Based on the whole egg remains found in China (egg length of ca. 19 cm; [41]), these were smaller individuals than those laying *Macroelongatoolithus* eggs in the Mussentuchit (egg length of 26–30 cm length). Thus, we interpret the taxon to be smaller bodied than giant oviraptorosaurs, more similar to the length of eggs known for the oviraptorid (14–16 cm, [76]) and *Citipati* (18–19 cm, [67, 69]). Putatively, we suggest that at least two sympatric size classes of oviraptorosaur coexisted in the Mussentuchit.

Recognition of *Continuoolithus* in the Mussentuchit Member makes it the earliest Cretaceous record of this ootaxon and narrows the temporal gap in the record between the Santonian-age Milk River Formation (ca. 83 Ma; [52, 101]) and the earliest described *Continuoolithus* from the Tithonian-age Brushy Basin Member of the Morrison Formation in New Mexico [102]. Whole egg remains of *Continuoolithus* range in length from 9.5–12.3 cm [103, 104]. Inclusion within Elongatoolithidae suggests these eggs belong to yet another smaller oviraptorosaur taxon with a smaller body mass than in all known parent-egg associations for this clade: *Oviraptor* (egg length 14.3–16 cm, [69, 79]), and *Nemegtomaia* (egg length 14–16 cm, [76]). Combined with the records of *Macroelongatoolithus* and *Undulatoolithus*, we now have evidence for at least three sympatric putative oviraptorosaurs in the ecosystem represented by the Mussentuchit Member.

Spheroolithidae has been taxonomically affiliated with hadrosauroid dinosaurs, with a direct association of body fossils and eggs documented for the saurolophine *Maiasaura peeblesorum* and *Spheroolithus albertensis* eggshell in the Two Medicine Formation of Montana [103, 105, 106]. However, the phylogenetic extent of this parent taxon-ootaxon association remains uncertain because there is no evidence yet as to whether Spheroolithidae eggs were exclusively laid by hadrosaurids, hadrosauroids, or a broader group of Ornithopoda. The presence of *Spheroolithus* in the Cenomanian indicates that the ootaxon is not restricted to Hadrosauroidea because the Mussentuchit Member has only one currently described hadrosauroid, the early-diverging and abundant hadrosauromorph *Eolambia caroljonesa* [20, 21], which is the most parsimonious egg-layer based on the taxonomic affiliation of *S*. *albertensis*. We have two *Spheroolithus* oospecies in the Mussentuchit oofauna, and the affiliation of the more recently described *S*. *europaeus* to a putative egg-laying taxon is much less discussed. In the description of the holotype, Sellés et al. [51] mentioned six described and many indeterminate hadrosauroids from the Late Maastrichtian of the southern European archipelago [107–113]. Spheroolithidae are also described from Lower Cretacous units in Europe, particularly across the Maestrazgo Basin in Spain (*Guegoolithus* [63]). No hadrosauroids have yet been described from these horizons, but early-diverging Ornithopoda (*Gideonmantellia turolensis* [114]) and Iguanodontia (*Delapparentia turolensis* [115]) co-occur in the Lower Barremian Camarillas Formation with *Guegoolithus*.

In the Mussentuchit Member, the assemblage of Ornithischia is more similar to the Early Cretaceous deposits of Western Europe before the hadrosauroid diversification in North America in the Late Cretaceous [116]. Recent discoveries from NCSM and FMNH expeditions to the Mussentuchit Member reveal five clades of Neornithischia—Thescelosauridae (*Fona herzogae* [117], Rhadodontomorpha (*Iani smithi* [9]) and Hadrosauriformes (*Eolambia caroljonesa* [20, 21]), plus fragmentary ceratopsians and ankylosaurians—were simultaneously present during the early Late Cretaceous of North America. One or both of the *Spheroolithus* oospecies could have possible affiliation with these taxa—with the possible exception of ceratopsians that may have laid soft-shelled eggs [118]—but the occurrence of multiple *Spheroolithus* oospecies in the early Late Cretaceous suggests a non-exclusive relationship with hadrosauroids. This highlights again the importance of eggs and ichnofossils when describing paleoecological make-up, in addition to solidifying the understanding of dinosaur lineage evolution through the Cretaceous. Further investigation into the early occurrences of *Spheroolithus* could better untangle its complex oofamilial relationships [47, 63] and constrain its taxonomic affiliations.

*Mycomorphoolithus* represents the first non-dinosaurian eggshell described from the Cedar Mountain Formation and would be the youngest known example of the oogenus, extending the documented temporal range from the Berriasian to the Cenomanian. Moreno-Azanza et al. [56] putatively affiliated *M*. *kohringi* to non-eusuchian crocodylomorphs based on the high relative abundance of this clade in the Maestrazgo Basin of Spain and a lack of some diagnostic Krokolithidae characters in the eggshell. Eggs of extant crocodilians are thickest in *Alligator mississippiensis*, *C*. *porosus*, and *C*. *niloticus* at a maximum of 0.53 mm [119], leading to the inference that the thicker eggs of *Mycomorphoolithus* reflect a larger taxon (see Crocodylomorph Nesting).

### Mussentuchit Member paleoecology

The Mussentuchit Member preserves the highest documented oodiversity among the lowermost Upper Cretaceous units of the Western Interior Basin. We recognize at least five oogenera in the unit. The ootaxa present here are representative of multiple oviraptorosaur taxa (*Macroelongatoolithus*, *Undulatoolithus*, *Continuoolithus*, Elongatoolithidae indet.), ornithopods (*Spheroolithus*), and Mesoeucrocodylia (*Mycomorphoolithus*). Only a single ootaxon, represented by an egg pair and eggshell fragments (*Macroelongtoolithus carlylei* [25]), has been described from the Wayan Formation of Idaho (101.0 Ma ± 1.1 to 97.5 Ma ± 2.0 Ma [120, 121] and another in the older Little Sheep Mudstone Member (ca. 110–115 Ma [122]) of the Cloverly Formation of Wyoming (*‘Deinonychus antirrhopus’* egg [82]).

**Fig 7:**
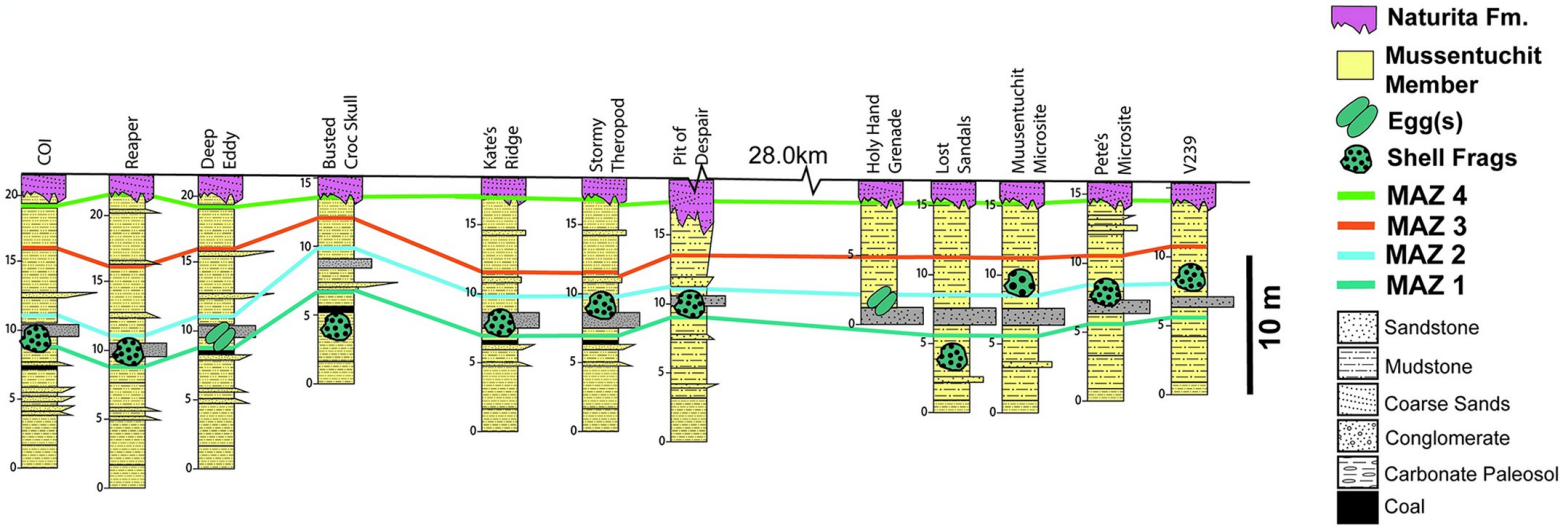
Stratigraphic sections for the Mussentuchit Member. This includes localities where either whole eggs or isolated fragments were recovered. MAZ = Mussentuchit Ash Zone. One of each oogenus is at least seen between the first two ash horizons MAZ1 and MAZ2, a range of less than 100 kyr.

Oodiversity of the Mussentuchit Member is comparable to some of the Campanian and Maastrichtian age dinosaur-bearing units of the Western Interior Basin (Table 1). The most diverse unit with a similar timeframe currently is the Campanian-age Kaiparowits Formation with eight recognized oospecies. Eggshell from the Kaiparowits Formation represents a temporal window of over two million years based on sandine Ar/Ar (ca. 76.46–74.69 Ma [123]) and detrital zircon U-Pb radiometic geochronology (as late as 73.10 Ma ± 1.20 Ma [124]), with multiple ootaxa occurring at the same locality [55]. The oogenera we document from the Mussentuchit Member are represented from at least one locality between MAZ1 (99.49 Ma ± 0.05) and MAZ2 (99.40 Ma ± 0.07; Fig 7), constraining the temporal window of the total oodiversity we describe in this unit to ca. 89,000 years.

**Fig 8:**
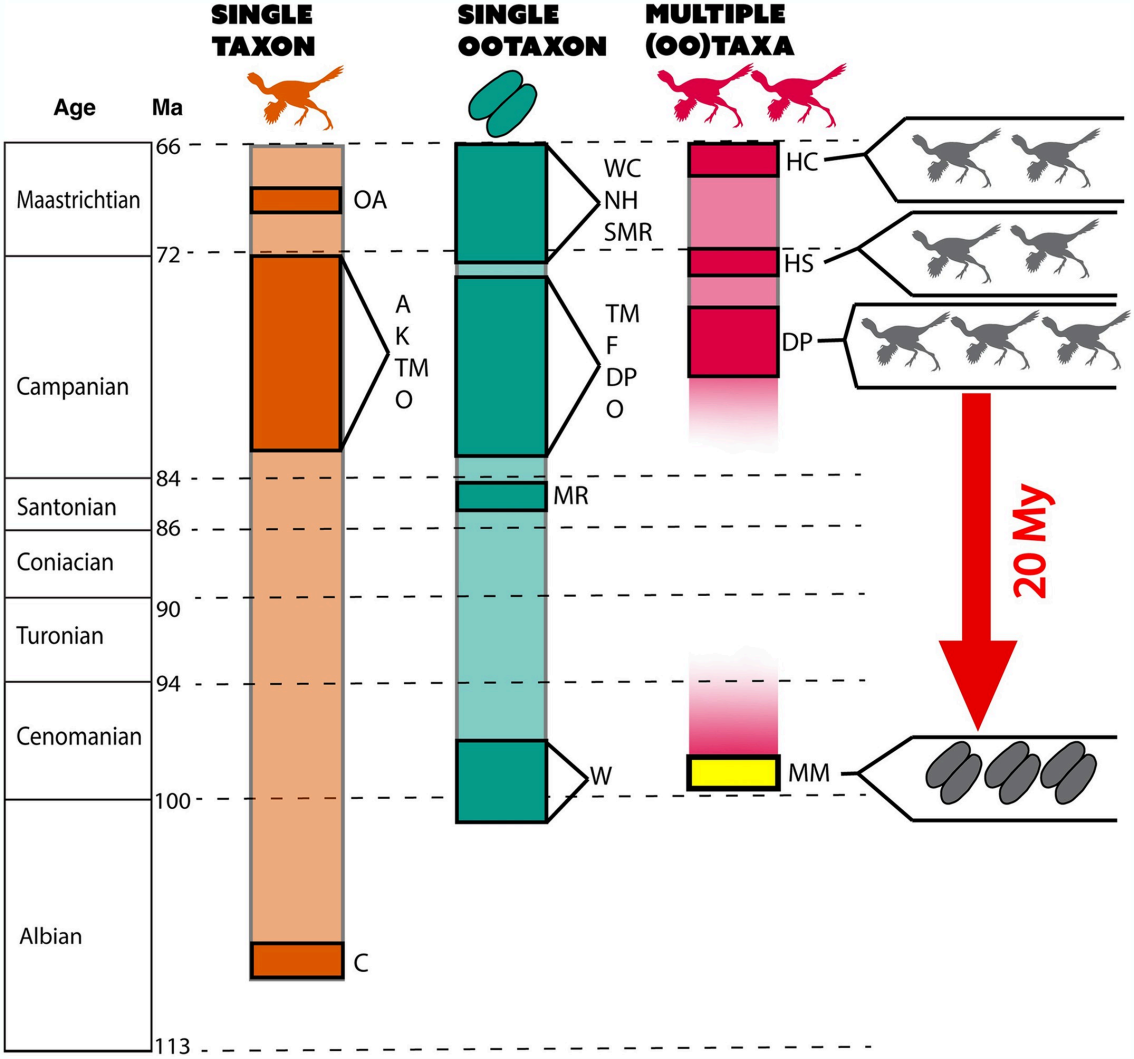
The fossil record of North American oviraptorosaurs during the middle to Late Cretaceous, as represented by body fossils and Elongatoolithidae. The three known oogenera of Elongatoolithidae in the Mussentuchit Member (highlighted in yellow) close a 20 million year ghost lineage of co-occurring oviraptorosaurs, where the previous oldest known co-occurrence was in the Campanian-age Dinosaur Park Formation. The Mussentuchit assemblage is also the first known co-occurrence of Elongatoolithidae in North America. A = Aguja Fm.; C = Cloverly Fm.; DP = Dinosaur Park Formation; F = Fruitland Fm.; HC = Hell Creek Fm.; HS = Horseshoe Canyon Fm.; K = Kaiparowits Fm.; MM = Mussentuchit Member (Cedar Mountain Fm.); MR = Milk River Fm.; NH = North Horn Fm.; O = Oldman Fm.; OA = Ojo Alamo Fm.; TM = Two Medicine Fm.; W = Wayan Fm.; WC = Willow Creek Fm.

The next oldest multiple eggshell-bearing unit in the Western Interior Basin is the Upper Santonian Milk River Formation [52], from which four theropod and one ornithopod ootaxa are documented. The lack of reported ootaxa between the Cenomanian and Santonian is likely due to a depauperate fossil record in the Turonian and Coniacian ages [16]. Zelenitsky et al. [52] noted the thinness of the Milk River Formation eggshell versus Campanian and Maastrichtian eggshell (although see Voris et al. [42]); conversely, we describe remarkably thick eggshells. In this study, we measured eggshell thickness digitally to remove uncertainty regarding embryonic age variation or dissolution of mammillae [125]; we only recorded thickness in fragments where the nucleation sites of mammillae were visible in radial cross-section. We note that measurement using calipers without assessing the presence or absence of complete mammillae, as conducted in previous studies, could bias results towards lower overall shell thickness. The *Continuoolithus* eggshells are on average 0.43 mm in the Milk River Formation, and 1.42 mm in the Mussentuchit; *Spheroolithus* similarly are on average 0.56 mm and 0.97 mm, respectively. The Milk River Formation is dominated (82% of all eggshell) by *Spheroolithus* eggshell, whereas the Mussentuchit is mostly *Macroelongatoolithus*, a record of colossal oviraptorosaurs (67% of fragments in this study, >1,500 fragments in Zelenitsky et al. [32]). Though the percentage of ootaxa does not reflect the percentage of body taxa in a given assemblage, the stable chemical composition of eggshell compared to body fossils across a range of environmental conditions during diagenesis could indicate more accurate ecosystem make-up than from body fossils alone [126]. *Macroelongatoolithus* do not appear in the Western Interior Basin fossil record after the Cenomanian, despite the persistence of mid-sized caenagnathids and *Continuoolithus* in Upper Cretaceous formations (Fig 8), suggesting the colossal oviraptorosaurs of the early Late Cretaceous may have gone extinct in North America before the Santonian. Nonetheless, they persisted through to the end of the Cretaceous in Asia [36, 97].

The earliest record of Prismatoolithidae, the oofamily associated with Troodontidae [127–129], is the Milk River Formation. There are records of Troodontidae in North America before the Santonian as far back as the Tithonian-age Morrison Formation (*Hesperornithoides* [130]; *Koparion* [131]), and isolated troodontid teeth in the Cenomanian from the Mussentuchit Member [2, 5, 132], the Naturita Formation [133], and the Lewisville Formation [134]. However, the lack of Prismatoolithidae from the Mussentuchit Member ooassemblage is notable given the preservation of isolated teeth, the expanded oodiversity presented in this study, and the prevalence of Prismatoolithidae in the Late Cretaceous of the Western Interior Basin [42, 43, 50, 52, 87, 90, 129, 135, 136]. The recent re-interpretation of the Mussentuchit Member as a paralic succession with a strong influence of brackish groundwater [38] is different from the Late Cretaceous prismatoolithid-bearing units that are predominantly alluvial plains and braided river systems [43, 52, 90] (although see Jackson and Varricchio) [50]. The topic of how environmental differences reflect differences in the nesting habits of the fauna here and the Late Cretaceous generally warrants future investigation.

The Mussentuchit Member records the earliest occurrence of the oogenus *Spheroolithus* in North America; the previous oldest known was from the Lower Campanian Two Medicine Formation of Montana [50]. The occurrence of *S*. *albertensis* here bridges a gap in the North American fossil record of more than 20 million years. It is also consistent with the diversification of the hadrosauroid clade with a recognized association to this ootaxon [63, 103, 105, 106], which came to prominence in North America during the early Late Cretaceous. Of note is the difference in porosity between the two oospecies of *Spheroolithus*, reported in Sellés et al. [51] and corroborated by the eggshells we describe in this study. *S*. *albertensis* has a much higher pore density (161 pores per cm^2^ [137]) than *S*. *europaeus* 48 pores per cm^2^ [51]), indicating lower overall porosity. Tanaka and colleagues [138] infer that differences in eggshell porosity in archosaurs reflect open or closed nesting modes. However, their data do not offer nesting states for hadrosauroids, much less insight into the higher resolution variation between oospecies. It is possible that *Spheroolithus* oospecies differed in porosity due to nesting modes, or differences in vapor conductance [137], thus justifying future investigation.

Ootaxa add valuable insight into the faunal make-up of the Mussentuchit and a greater understanding of the transitional state of the fauna during the early Late Cretaceous. However, they do not capture true dinosaur diversity in Cenomanian North America as no ootaxa is yet described for some of the last known allosauroids or sauropods as well as the immigrating tyrannosauroids, ceratopsids, and non-oviraptorosaurian maniraptorans [7–9, 139].

### North American oviraptorosaur distribution

The Mussentuchit Member is a window into the early radiation of Asian-immigrant fauna that proliferated in the early Late Cretaceous of North America [2, 7, 8, 19], a trend now corroborated with oological data. All theropod eggshell from the Mussentuchit Member described herein is attributable to Oviraptorosauria, a clade that originated in Asia and migrated to North America—possibly on multiple occasions—during EKLInE (e.g. [2, 8, 17–19]. The new data we have presented here significantly impact our understanding of oviraptorosaur distribution of the Late Cretaceous in North America. Eggshell is crucial to understanding oviraptorosaurian occurrence as this edentulous clade does not shed teeth that could otherwise be found in microsites. The latest Cretaceous oviraptorosaur record in North America is largely uninterrupted (at the resolution of whole formations) through the Campano-Maastrichtian (Fig 8), particularly when oviraptorosaurian ootaxa are included. Across this uppermost Cretaceous timespan, there are ten described oviraptorosaur species (all of which are Caenagnathidae [140, 141]) ranging from Alberta in the north [140] to Coahuila, Mexico in the south [142]. There is a ca. 30 million year gap in the body fossil record between these taxa and the small-bodied taxon from the Cloverly Formation of Wyoming [24], *Microvenator celer*. This large temporal gap diminishes when we incorporate Elongatoolithidae as a proxy for Oviraptorosauria. Elongatoolithid eggshell from the Milk River Formation [52], the Wayan Formation [25], and the Mussentuchit Member of the Cedar Mountain Formation [26, 33, this study] suggests the persistence of oviraptorosaurs in North America throughout most of the Cretaceous.

Moreover, the Mussentuchit Member preserves at least three co-occurring elongatoolithid ootaxa, indicating the presence of at least three coexisting oviraptorosaurian taxa. This is not surprising; multiple elongatoolithid oogenera (*Elongatoolithus*, *Macroelongatoolithus*, and *Paraelongatoolithus*) are known to co-occur in the Chichengshan Formation of Zhejiang, China [36], eight oviraptorosaur species are described in the Nemegt Formation of Mongolia [143], and seven species from the Nanxiong Formation of China [144]. Although the Late Cretaceous body fossil record for oviraptorosaurs is diverse, co-occurrence in North America of multiple oviraptorosaurian taxa is rarely seen. Only three formations in the Upper Cretaceous yield multiple oviraptorosaurian taxa: the Dinosaur Park Formation (n = 3 [143]); the Horseshoe Canyon Formation (n = 2 [145]); and the Hell Creek Formation (n = 2 [141]), and the tentative co-occurrence of two taxa in the Frenchman Formation [146]. The Mussentuchit Member’s three elongatoolithid ootaxa are the first documentation of multiple species of co-occurring North American oviraptorosaurians solely from ootaxa. These fill a vital gap in our understanding of mid-Cretaceous oviraptorosaurian paleobiogeography and paleoecology. The occurrence of large elongatoolithid eggs, and large-bodied caenagnathids, in both the Wayan and Cedar Mountain Formations pre-dates similarly sized Asian taxa such as *Gigantoraptor* [93], aged at 95.8 ± 6.2 Ma [147] or *Beibeilong* [78] from the middle Turonian to middle Campanian Gaogou Formation [41]. Moreover, with at least three ootaxa present in the early Cenomanian of Utah and Idaho, we can infer a larger niche occupation than previously suggested for North American oviraptorosaurs, and that would not be evident solely from body fossil evidence. It also bisects an approximately 20 million year gap of co-occurring oviraptorosaur taxa from before the deposition of the Dinosaur Park Formation, and is now the oldest known instance of multiple oviraptorosaurian taxa co-occurring in a North American formation.

### Crocodylomorph nesting

NCSM 35004 and 35005 represent the first crocodylomorph eggshell documented from the Mussentuchit Member, the first occurrence of *Mycomorphoolithus kohringi* outside of Europe, and the youngest known fossil attributable to this ootaxon by about 30 million years. Moreover, they provide direct evidence of non-dinosaurian macrovertebrate reproduction in the ecosystem. The eggshell found here represents that of one or more mesoeucrocodylian species [56] which are not uncommon in the member as body fossils [2, 5]. Crocodylian material should be expected from this location given the proximal position to open water aquatic systems [38], and teeth are found consistently in relatively high abundance in both quarries and at microsites [5, 21, 148]. It is likely that shed teeth are overrepresented and body fossils underrepresented given the taphonomic regime [5]. These teeth are nearly exclusively attributable to stem mesoeucrocodylian taxa: bernissartiids, atoposaurids, pholidosaurids, and *Dakotasuchus*, a phylogenetically unstable mesoeucrocodylian (goniopholidid in Frederickson et al. [149]; pholidosaurid in Jouve and Jalil [150]) for which the coracoid, dorsal vertebrae, and osteoderms are also described [149]. Cifelli et al. [2] also report the teeth of the teleosaurid *Machimosaurus*, that the authors suggest as a ‘probable last occurrence’ with most teleosaurid fossils known from the Jurassic. *Woodbinesuchus* (goniopholidid in Frederickson et al. [149]; pholidosaurid in Jouve and Jalil [150]) and *Terminonaris* (Pholidosauridae [150–152]) are described from the penecontemporaneous Woodbine Formation of Texas.

Environments in Europe with *Mycomorphoolithus* have yielded similar levels of diversity of stem Mesoeucrocodylia from body fossils: abundant material comes from Spain, including the holotype locality of La Cantalera [56] and Vadillos-1. The Purbeck Limestone of England has also produced material reinterpreted as *Mycomorphoolithus* sp. following reinterpretation by Moreno-Azanza et al. [56]. La Cantalera, an exceptionally diverse European locality in the Blesa Formation at the Hauterivian-Barremian boundary, represents a marshy environment with periodic droughts and non-permanent water [153, 154]. It preserves Bernissartiidae and Goniopholididae teeth, as in the Mussentuchit Member, plus cf. *Theriosuchus* and cf. *Lisboasaurus* teeth. Vadillos-1 of the El Collado Formation, an alluvial-palustrine muddy floodplain [155], preserves the teeth of Bernissartiidae, Atoposauridae, and Goniopholididae (taxa consistent with the Mussentuchit) as well as the deeply-nested hylaeochampsid *Unasuchus* [134], in addition to *Mycomorphoolithus*.

The Purbeck Limestone Group in southern England is older (Berriasian) and predominantly offshore [156]. The eggshell referred to *Mycomorphoolithus* (‘Type 1’ [57]; ‘Type 1 and Type 2’ [58], sensu Moreno-Azanza et al. [56]) is from the Worbarrow Tout Member of the Lulworth Formation, described as chert-rich micrites [156]. Two crocodylians are known from body fossils recovered from the Purbeck Limestone Group: the goniopholidid *Goniopholis kiplingi* [157], and the pholidosaurid *Pholidosaurus purbeckensis* [158]. Both of these clades have been identified in the Mussentuchit Member based on isolated teeth.

**Fig 9:**
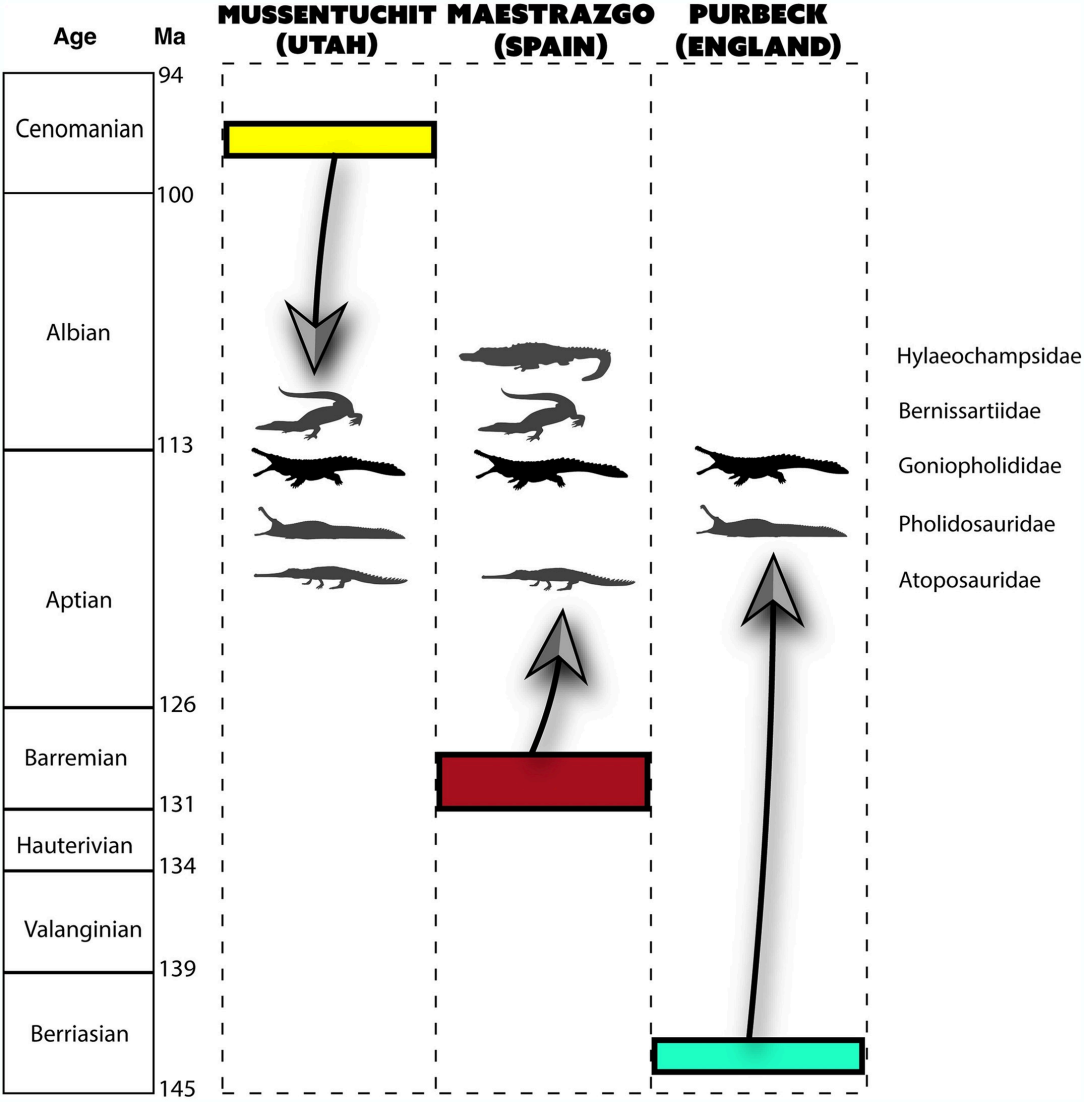
The known fossil-bearing strata of *Mycomorphoolithus* and co-occurring mesoeucrocodilians that have been posited as potential egg-layers. The discovery of *Mycomorphoolithus* in the Mussentuchit extends the temporal and spatial range of this oogenus from Early Cretaceous Europe across into Late Cretaceous North America. Goniopholididae are the only known clade with representation in all three units with *Mycomorphoolithus*.

Taxa from the Mussentuchit belong to clades that, in North America, range from 0.6 m long *Bernissartia* to 6 m long *Terminonaris*; body size from the described *Dakotasuchus* in the Mussentuchit has been estimated at 3.7–5.4 m [149]. Any clades referenced above could represent the putative egg layer with no indication of egg size to constrain the speculation or direct association of osteological fossils with eggshell. It may be worth noting that the only taxon cited in each of the three regions with *Mycomorphoolithus* are goniopholidids (Fig 9). Still, considering the isolated nature of eggshell and teeth found along with the instability of the taxa within Goniopholididae and Pholidosauridae, we do not ascribe an association.

## Conclusions

Our results fill a major ootaxonomic knowledge gap among mid-Cretaceous assemblages of Western North America, which lag behind those of the Late Cretaceous. Based on our study, the Mussentuchit ooassemblage is currently the most diverse known prior to the Campanian. Due to the newly formulated temporal framework, at least six archosaurian ootaxa are represented from a ~89,000-year window. Three of these provide novel insights for the understanding of oviraptorosaur spatiotemporal distribution and diversity within the Late Cretaceous Western Interior Basin. We document the presence of at least three differently sized co-occurring oviraptorosaurs, of varying size classes, in the Mussentuchit assemblage. These data bridge a 20 million year gap in the fossil record of co-occurring North American oviraptorosaurs, another 15 million years in the record of *Spheroolithus* in North America, and a 30 million-year gap for the crocodylian ootaxon *Mycomorphoolithus*. Eggshell data are particularly crucial to understanding broader paleoenvironmental questions. They provide data points beyond body fossils alone and a fascinating window into the behavioral ecology of these taxa.

## Supporting information

S1 Table Full dataset of microstructural measurements of eggshell fragments. (CSV)
